# Developing Courses of Spanish for Specific Purposes in Agriculture to Bridge the Communication Gap Between the Hispanic Workforce and English-Speaking Veterinary and Animal Sciences Students

**DOI:** 10.3390/ani14243639

**Published:** 2024-12-17

**Authors:** Leonor Salazar, Allen Jimena Martinez Aguiriano, Silvana Pietrosemoli, Arlene Garcia

**Affiliations:** 1 Texas Tech University School of Veterinary Medicine, Amarillo, TX 79106, USA; leonor.salazar@ttu.edu (L.S.); allemart@ttu.edu (A.J.M.A.); 2 Department of Animal Science, College of Agriculture and Life Sciences, North Carolina State University, Raleigh, NC 27695, USA; silvana_pietrosemoli@ncsu.edu

**Keywords:** development of courses, animal science students, veterinary science students, animal welfare, Spanish for Specific Purposes in Agriculture, Hispanic workforce

## Abstract

This study addresses the integration of Spanish for Specific Purposes in Agriculture (SSPA) into the curricula of English-speaking veterinary and animal sciences students at three collaborating universities (Texas Tech University School of Veterinary Medicine, North Carolina State University, and Tarleton State University), to bridge communication gaps in agricultural settings. We designed and implemented three SSPA courses focused on key areas such as animal health, welfare, and food safety, using insights from industry professionals. The courses incorporated contextualized language instruction, including vocabulary, grammar, and practical communication scenarios relevant to the field. Teaching materials including interactive resources and metacognitive strategies were developed to enhance learning and engagement. The results showed that while the courses improved students’ communication skills, challenges such as technical issues and balancing academic commitments required adjustments to course delivery. Significant differences in performance were observed between institutions, with a considerable impact on the course completion rates and final test scores. The study concludes that SSPA courses significantly enhance the ability of veterinary and animal sciences students to effectively communicate with the Hispanic workforce, providing a model for similar language integration efforts in specialized fields.

## Simple Summary

The increasing diversity of the agricultural workforce has created a pressing need to address the communication gap between English-speaking veterinarians/animal science professionals and the growing Spanish-speaking Hispanic population employed in these industries. To effectively meet the needs of diverse populations in agricultural settings, it is crucial that veterinary and animal science programs develop a workforce that can navigate cross-cultural communication. One promising approach to bridging this gap is the integration of Spanish for Specific Purposes in Agriculture (SSPA) in veterinary medicine and animal science programs focused on the unique language and cultural needs of the agricultural sector. Implementing SSPA courses within veterinary and animal sciences curricula can provide students with the targeted language skills and cultural competence necessary to communicate effectively with the Spanish-speaking workforce. This approach is designed to equip students with the specific communication skills required for their future careers, ensuring they are prepared to thrive in their chosen professions by developing language skills needed to communicate with the Hispanic workforce.

## 1. Introduction

The U.S. agriculture industry has experienced significant growth in terms of the Hispanic workforce over the past two decades. As of 2023, Hispanics represent 18.8% of the 161,037,000 million individuals in the U.S. workforce. Of these, 25.4% are employed in agriculture, forestry, fishing, and hunting, and 22.2% are involved in animal production and aquaculture [1]. Despite this substantial representation, many Hispanic workers have limited English proficiency, leading to communication barriers with English-speaking farm personnel and animal professionals. These communication gaps have been reported to adversely affect farm operations, animal productivity, and welfare in the United States [2,3]. Veterinary and animal science professionals play a crucial role in educating and training animal caretakers and disseminating vital information about animal management and care. Effective communication is essential to ensure animal health, welfare, and food safety. However, linguistic diversity and English limitations among the workforce complicate these interactions, potentially impacting agricultural production and food safety [4]. Miscommunication can also lead to the improper handling of livestock, increasing safety risks and animal distress [5]. 

The sustained employment of immigrant labor in the U.S. agriculture sector further exacerbates these challenges, as Spanish-speaking animal caretakers are increasingly occupying roles traditionally held by English speakers [6,7]. Miscommunication between English-speaking management and Spanish-speaking employees can lead to misinterpretation and affect the accuracy of animal diagnoses. Additionally, English-speaking employees often hold higher management positions, while non-English-speaking workers are typically employed in roles directly dealing with animals [8].

Given the expansion of animal production businesses and the growing need for Hispanic labor, developing Spanish communicative competence among English-speaking animal professionals has become a priority. Despite this, efforts to bridge the communication gap between English-speaking personnel and non-English-speaking animal caretakers have remained limited.

This paper aims to describe the design and implementation of three courses of Spanish for Specific Purposes in Agriculture (SSPA) aimed at enhancing Spanish communicative skills among veterinary medicine and animal science students. 

These courses are designed to provide students with the necessary language skills to achieve a functional and technical use of the Spanish language, focusing on field-specific communication, to bridge the gap between English-speaking personnel and Spanish-speaking animal caretakers. This paper represents the second phase of a U.S. Department of Agriculture capacity-building grant awarded for a research project titled “Developing Spanish Communicative Competence among Veterinary and Animal Science Students to Improve U.S. Agriculture”. This multi-institutional initiative involves Texas Tech University School of Veterinary Medicine (TTU SVM), North Carolina State University (NCSU), Tarleton State University (TSU), and Universidad Nacional Autónoma de México (UNAM).

The initial phase involved analyzing communication needs through a survey administered to English-speaking animal professionals, exploring their language needs when interacting with Spanish-speaking caretakers [9]. The survey results led to the development of the SSPA courses designed and discussed in this paper.

### 1.1. Language for Specific Purposes

Language for Specific Purposes (LSP) emerged alongside the communicative approach to language teaching in the 1970s. LSP focuses on tailoring language instruction to meet specific needs in professional or academic contexts [10,11,12]. Needs analysis (NA) is crucial in LSP course design and involves the use of methods such as surveys and interviews to identify learners’ linguistic requirements and contexts. This process informs the selection of the relevant language skills, tasks, and materials to be used in the design and implementation of such courses. 

Modern constructivist views emphasize student-centered learning, where the course design accounts for learners’ prior knowledge and experiences [13]. This approach prioritizes tailoring syllabi to specific communicative functions and contexts, integrating various language skills and sub-skills relevant to learners’ fields of specialization [14,15]. 

### 1.2. Types of LSP Courses

Originally, the term used for LSP courses was English for Specific Purposes (ESP), since the first courses focusing on the specific needs of the participants were mostly in English. Over time, the more general term of Language for Specific Purposes was adopted. However, most of the literature in the 1970s and onwards still uses the term ESP. As ESP courses and learner-centered approaches gained momentum, different categories emerged based on the purposes of the course and the field of study. Mumby [16] established two general ESP categories: English for Occupational Purposes and English for Academic Purposes. Hutchinson and Waters [17], in their ‘Tree of English Language Teaching’, mentioned three types of ESP courses: English for Science and Technology, English for Business and Economics, and English for Social Studies. They also proposed that ESP should be considered “an approach to language teaching in which all decisions as to content and method are based on the learner’s reason for learning” [17]. Additionally, Evans and St. John [18] classified ESP courses by professional area, including English for Academic Purposes and additional categories such as English for Science and Technology, English for Medical Purposes, English for Legal Purposes, and English for Management, Finance, and Economics. They subdivided English for Occupational Purposes into two main categories: English for Professional Purposes and English for Vocational Purposes.

An earlier classification was also suggested by Strevens [19], who pointed out the distinction between English for Science and Technology and other types of English courses. Both groups can be further classified into educational or occupational groups. What is relevant in Strevens [19] view is the distinction between in-training and post-training English for Occupational Purposes courses, depending on the time of instruction; that is, whether language instruction takes place at the same time the participants are being trained for the job, or after graduation when they have already joined the workforce or are practicing professionals. 

In the case at hand, our SSPA courses are aimed at promoting communication between English-speaking veterinary and animal sciences students and the Hispanic workforce in animal farm settings. According to the classification parameters discussed above, these are courses of Spanish for occupational purposes, which are pre-occupational (or in-training) and field-oriented, for which it is essential to carry out a needs analysis prior to designing the syllabus and the materials to determine the competencies needed for communication in Spanish and other relevant information for course design and implementation.

### 1.3. Current Trends in LSP

Recent LSP research addresses several key areas: contemporary teaching practices and course design, learner and teacher challenges, future research directions, cross-cultural communication and intercultural awareness, and the impact of virtual learning. Internationally, there is growing research on and implementation of LSP courses, including in military settings. Methodologies include language as a medium of instruction, integrated content and language-integrated learning [20]. The combination of these approaches, along with the integration of cognitive and metacognitive strategies, reflects the modern understanding of language teaching.

LSP has gained popularity over the years, with renovated views of language curricula stemming from the emphasis on interdisciplinary connections and cultural awareness. Risner and Spaine-Long [21] report that the first International Symposium of Language for Specific Purposes was held at the University of Alabama at Birmingham in 2012 and every two years since then at different universities across the United States. This is an indicator of the increasing community of LSP educators, the ongoing preparation of teachers and practitioners, and the relevance and growth of LSP courses in the academic environment [22].

Even the military has realized the importance of LSP training, as LSP courses have been implemented in the engineering programs at the United States Military Academy at West Point [23,24,25]. In preparing cadets for their military engagements around the world, the Department of Foreign Languages of the Military Academy implements learner-centered and career-oriented LSP courses, where military-related LSP content is embedded within classroom instruction.

It is also interesting to see how across Europe, and especially in northern and eastern European countries, there has been an increase in research and publications on LSP, as well as in the number of LSP courses implemented at the graduate and undergraduate levels. Particular relevance has been given to LSP as a means to learn a professional foreign language in the agriculture and agro-engineering fields [20,25,26,27,28,29]. 

### 1.4. Spanish for Specific Purposes in Veterinary Science and Agriculture

While LSP research is well developed, specific studies on Spanish for Specific Purposes (SSP) in agricultural contexts are limited. Most of the existing literature focuses on English for Specific Purposes (ESP) and general occupational language training, with few studies addressing SSP in the veterinary and agricultural fields. Efforts to use SSP in veterinary sciences, such as those at Colorado State University and Texas A&M University, demonstrate growing interest but also highlight the challenges in integrating language training in veterinary curriculum [30,31]. These programs face difficulties in balancing language instruction with the demands of veterinary education, suggesting the need for more tailored and integrated SSP courses. 

We have already pointed out that the inability to communicate in a foreign language in the work field harms the successful exchange of information and yields potentially negative outcomes for interlocutors. Considering that fewer than 20% of veterinary students in the United States can provide medical information in Spanish to limited English-proficient Spanish-speaking clients, it is important to overcome this language barrier, which limits access to knowledge, hinders communication, and affects work performance. In addition, in a multilingual society, such as the United States, where most of the workforce is of Hispanic origin, communication between English-speaking professionals and the Hispanic workforce must be fostered. In this context, SSP in-training courses in veterinary medicine programs are growing and evolving, and appropriate methods for course design and implementation must be further researched and developed.

Given that veterinarians often work with culturally and linguistically diverse teams [32], the development of SSP programs in agricultural and veterinary education is fundamental to ensure that our future workforce develops the required communication skills to discuss topics such as animal health, welfare, production, and food safety [9]. Prior research experience, which deals with SSP in veterinary medicine, stresses the need for discipline-specific Spanish language training for veterinary students [33]. They advocate for an interdisciplinary approach to Spanish syllabus design and discuss the challenges of implementing a foreign language program in the already demanding veterinary curricula.

Forehand, Zeller, and Frey [33] described the results of an experience at Colorado State University. An anonymous survey was administered to students of veterinary medicine programs throughout the U.S. to collect information on previous Spanish language learning experiences, students’ interest in engaging in Spanish coursework specifically designed for the veterinary field, their motivations, desires, and expectations, and their learning preferences. The results indicate that interest in Spanish courses in the veterinary field is high, most students are willing to participate in the courses, and most respondents’ previous experiences with learning Spanish only occurred during high school. The information gathered could guide the curricular design decisions for Spanish for Veterinarians Language Program (SVLP). The program includes SSP courses at beginner and intermediate levels aimed at increasing the Spanish proficiency levels of veterinary students, enabling them to communicate with their prospective Spanish-speaking clients. 

In the fall of 2013, Texas A&M University College of Veterinary Medicine and Biomedical Sciences introduced medical Spanish during the second year of the curriculum program for veterinary medicine students. The purpose was to respond to the needs of prospective veterinarians to prepare for the changing demographics and the growth of the Hispanic population in the United States. It was a five-week course composed of lectures and group learning to provide a basic medical vocabulary and a limited number of useful phrases [31]. The researchers outlined the implementation of the foreign language course as a curricular component of cultural competency, including their pedagogical approaches to curricular design and a discussion of the successes and challenges. 

A nationwide study conducted in 2013 revealed that only 2.1% of U.S. veterinary students spoke Spanish as their first language, and just 16.4% reported being conversant in Spanish. This same study showed that while 47% of the students felt able to communicate socially with Spanish speakers, fewer than 8% felt prepared to provide medical information to Spanish-speaking clients [34].

Learning or acquiring a second language is a complex process that varies from one individual to another. It is neither a uniform nor a predictable process. Moreover, it is the result of the interrelation between internal and external factors that influence one another. The former include age, gender, intrinsic and extrinsic motivation, aptitude, prior knowledge, learning mechanisms/styles, personality, and cognitive style, while the latter comprise social elements that determine the social disposition of the learner for learning a second language, such as social identity, social class, ethnicity, teaching strategies, and language input [35,36,37]. In addition to these factors, another study [38] states that working memory, metacognition, cognitive control, and autonomy also influence foreign language learning and acquisition, while another study [37] points out that other intervening factors include native language, attention, memory, problem-solving, perceptual abilities, social interactions, collaborative learning, and cultural contexts. 

### 1.5. Challenges and Opportunities

Key challenges in SSP course implementation include integrating language training into the existing curriculum and addressing specific disciplinary needs. Gaps in research indicate there is a need for a more focused development of SSP courses in the veterinary and agricultural fields. Our study addresses these gaps by detailing the design and implementation of SSPA courses aimed at enhancing Spanish communicative competence among veterinary and animal science students. After conducting a needs analysis with a field-oriented approach, designing and implementing Spanish courses for Specific Purposes in Agriculture (SSPA), we aimed to bridge the communication gap between English-speaking professionals and Spanish-speaking animal caretakers.

## 2. Materials and Methods

All work was approved by the Human Research Protection Program of Texas Tech University (IRB 2021-250) before data collection.

### 2.1. Development of the Courses

#### 2.1.1. Needs Assessment

The development of the Spanish for Specific Purposes in Agriculture (SSPA) courses was guided by a comprehensive needs assessment. An anonymous online survey was conducted with animal professionals—including veterinarians, animal scientists, farm owners, managers, nutritionists, agribusiness consultants, trainers, and professors—to identify communication challenges and language needs when interacting with Spanish-speaking animal caretakers [9].

#### 2.1.2. Course Objectives

The objectives of the SSPA courses were clearly defined to enhance specific competencies and skills relevant to veterinary and animal science based on the survey results. 

#### 2.1.3. Course Design

The results derived from the survey in the first stage of this work were used for designing three syllabi and developing the instructional materials for three courses of Spanish for Specific Purposes in Agriculture (SSPA) [9]. Such courses were addressed to veterinary and animal science students with low-intermediate (SSPA1), intermediate (SSPA2), and high-intermediate (SSPA3) levels of proficiency in Spanish.

Initially, a draft of the course was developed by a team of experts in linguistics, animal welfare, and animal production. This interdisciplinary team was tasked with creating a comprehensive outline that addressed the essential topics and vocabulary necessary for effective communication between English-speaking veterinary/animal science students and Spanish-speaking farm workers. To enhance the didactic quality of the course’s materials, a graphic designer was recruited. The designer’s role was to create visually appealing and user-friendly content, which facilitated the ease of understanding and engagement. The design process emphasized clarity and accessibility, ensuring that the lessons were not only informative but also appealing and engaging for learners.

Following the creation of draft content and visual materials, a review was conducted by a group of bilingual professionals. This review aimed to verify the accuracy and cultural appropriateness of the course content. Bilingual experts provided feedback to refine and ensure the correctness of the lessons, thus enhancing the overall quality and effectiveness of the instructional materials. This iterative process of development and review is critical for designing courses that are both pedagogically sound and practically relevant for veterinary and animal science students.

#### 2.1.4. Advertisement and Recruitment Process

A targeted outreach strategy was implemented to recruit candidate students from TTU, NCSU, and TSU. Informational flyers were distributed to departments with students with potential interest in the program. These flyers provided essential details, as well as the requirements and benefits of the program (available in Supplementary Materials). The candidates had to be willing to develop their communication skills in the Spanish language to effectively communicate with animal caretakers, livestock handlers, staff members, and other Spanish-speaking workers in typical farm settings. Each flyer included the email address of the Principal Investigator (PI) for further inquiries. When inquiries were received, the students were provided with detailed instructions on the next steps, including the need to take an online Spanish Placement Exam (SPLEX) to determine proficiency levels and appropriate course placement. Given that the goal was to reach an audience with a low–intermediate proficiency level in Spanish, the minimum score required for the SPLEX was 300, which corresponded to this level. Participants were students from veterinary medicine and animal science programs at TTU, NCSU, and TSU; therefore, they had some competence in agricultural-related topics.

### 2.2. Course Implementation

SSPA courses were delivered through an online platform with synchronous sessions to accommodate the diverse schedules of the participating students. This format was chosen to provide flexibility and facilitate real-time interaction between students and instructors. SSPA 1 was conducted in fall 2022, with sessions held twice a week for 1.5 h. SSPA 2 followed in spring 2023 with a similar schedule, and SSPA 3 was offered in summer 2023 with more frequent 2 h sessions four times a week. This approach enabled consistent engagement and regular feedback. A periodic assessment of students’ progress was conducted through online formal (written) testing at the end of each training unit. Students were also required to take a final test at the end of each SSPA course. 

#### 2.2.1. Statistical Analysis

The scores for the Spanish Placement Exam were collected when the authors requested that the students send back their results to determine whether they were eligible to join the program. Information on score performance was collected during the process with the support of the instructor. Once the authors had all the relevant information, the data were organized and managed in Microsoft Excel^®^ for Microsoft 365 MSO (Version 240). Data were then imported into R statistical software (Version 4.3.2), which facilitated the exploration of patterns and trends in student performance.

##### Spanish Placement Exam

Data on performance were collected through the Spanish Placement Exam (SPLEX) for individuals categorized by institution (Texas Tech University [TTU], North Carolina State University [NCSU], and Tarleton State University [TSU]). Scores were recorded following the selection process, and descriptive statistics were calculated, including the count, sum, average, and variance of the test scores.

To analyze differences in SPLEX scores based on institutional affiliation, a one-way Analysis of Variance (ANOVA) was employed. The dependent variable for this analysis was the SPLEX scores. Additionally, post hoc analyses using Tukey’s test, and the Bonferroni correction were conducted to identify specific differences among institutions.

##### Student’s Interest in the Program

The number of students who expressed interest in enrolling in the program, the number of students who completed the Spanish Placement Exam, and their performance on the exam were tracked. The results were summarized to identify the number of students who passed and their average scores. 

##### Student Performance in the SSPA Courses

Final scores were collected from each SSPA course after completion. To evaluate differences in performance across the three courses, a repeated measures ANOVA was conducted. Before the analysis, the Shapiro–Wilk and Jarque–Bera tests were performed to assess normality, and Levene’s test was used to evaluate the homogeneity of variance. Mauchly’s test was applied to check for sphericity, and Greenhouse–Geisser corrections were utilized as necessary. The data did not follow a normal distribution, and several outliers were identified. One outlier was removed from the NCSU group for SSPA 1. The Afex package (R statistical software, Version 4.3.2) was utilized, and the institution variable was converted into a factor.

##### Correlation Analysis

Before conducting a correlation analysis, the Shapiro–Wilk test was employed to assess normality. Given that all *p*-values were significantly less than 0.05, indicating a non-normal distribution, Spearman’s rank correlation was selected to assess the relationships between variables. A heatmap was generated to visualize the correlation results. A non-parametric test was then performed.

## 3. Results

### 3.1. Needs Assessment

The needs assessment survey results reported in our previous paper [9] revealed the need to be able to communicate in the oral medium for both oral receptive (listening) and oral productive (speaking) skills. Also, the ability to communicate with Spanish-speaking caretakers was important for conducting on-farm activities such as understanding descriptions of animal conditions, animal behavioral changes and disease symptoms, explaining animal management protocols, treatment administration, humane handling and restraint, euthanasia, and discussing record-keeping. 

### 3.2. Course Objectives

For SSPA 1 (low-intermediate), the focus was on foundational conversational skills and the basic grammatical structures necessary for introductory interactions with Spanish-speaking individuals. SSPA 2 (intermediate) aimed to build on these skills by introducing intermediate vocabulary and more complex grammatical constructs, whereas SSPA 3 (high-intermediate) targeted advanced proficiency, enabling students to effectively handle communication scenarios.

### 3.3. Curriculum Structure

The curriculum is structured into three progressive levels, each designed to address the competencies required at various stages of language proficiency. SSPA 1 concentrated on essential vocabulary and basic grammar through interactive lessons and role-playing exercises. SSPA 2 expanded on this foundation with intermediate-level content and more complex tasks. Finally, SSPA 3 included advanced discussions, specialized vocabulary, and real-life simulation scenarios relevant to veterinary and animal science practices. Each course incorporated a mix of pedagogical approaches, including task-based learning and communicative and information gap activities, to ensure a comprehensive and meaningful learning experience. 

### 3.4. Implementation of the Courses

#### 3.4.1. Courses Description

The selection of topics, language functions, contextualized settings, and communicative situations, with their corresponding key grammar and vocabulary items, stemmed from a thorough analysis of the communication needs as reported by professionals in the industry. Hence, the courses were specifically tailored to fulfill such realistic language needs.

The SSPA courses were all based on the premise that the ability to communicate in Spanish is a key factor in bridging the widely reported communication gap between English-speaking animal professionals and Hispanic on-farm animal caretakers. The courses are field-oriented since they are restricted to the specific discipline of agriculture; occupational because their content and the skills developed are intended to be used in actual work sites; and in-training since course participants were still enrolled in their academic programs and were being trained for prospective jobs. They comprise a range of topics, key vocabulary, and activities aimed at promoting communicative competence in Spanish in specific areas such as animal health, animal welfare, and food safety. Throughout the development of the courses, students gradually became equipped with the four language macro-skills (speaking, listening, reading, and writing) with an emphasis on the oral mode of the language to enable students to effectively communicate in the Spanish language in the framework of real-life on-farm settings.

An enriched teaching perspective supported the design of these courses. Their communicative nature is firmly rooted in the theoretical assumptions of communicative language teaching (CLT) coupled with general principles derived from the constructivist theory and the study of metacognitive processes. Students’ prior knowledge of agriculture-related topics, along with their current competence in Spanish, served as the basis for expanding their specialized vocabulary and developing new language skills. This blended and enhanced teaching approach was adopted to provide students with learning experiences that take advantage of their language abilities and their full cognitive potential.

#### 3.4.2. Teaching Strategies

The courses comprised a variety of teaching strategies and instructional materials that were student-centered. Although they addressed the four macro-skills of the language, emphasis was placed on the oral receptive and oral productive skills (listening and speaking) to align with the needs assessment results. Therefore, students were continuously prompted to use the macro-skills they were developing to actively take part in class discussions and interactions with peers. 

The teaching strategies fostered meaningful learning and were implemented to gradually fulfill the objectives set for each course by engaging the participants in contextualized active communication. They were designed to gradually help students build the language skills needed to communicate effectively and included pair interaction, group discussion, guided conversations, roleplays, information gap activities, listening comprehension exercises, identifying the main ideas and supporting details in audio/written texts, and inferring meaning from context. Other skills included predicting what other speakers might say, drilling, discussing the content of videos, interpreting diagrams and visual aids, summarizing information from written and oral sources, reporting facts, filling out forms, and giving specific presentations on farm protocols and audit topics. 

#### 3.4.3. Teacher’s Role/Scaffolding

Even though most of the classwork focused on students’ participation, the language instructor’s role was crucial for achieving the goals. The instructor was responsible for presenting the teaching materials, regularly engaging participants in contextualized discussions and interactions, monitoring student progress, orchestrating all of the teaching procedures, providing students with timely and appropriate assistance, and scaffolding to support meaningful learning experiences within a low-anxiety and friendly environment. The teacher’s continuous support served as a platform for students to gradually build the target language system. Students were always encouraged to interact with their classmates in simulated real-life conversations on the previously selected agriculture topics. Individual assistance and support were also available at all times to foster learner’s autonomy as they used the target language. 

#### 3.4.4. Teaching Materials

Three sets of teaching materials were carefully and specifically designed for the SSPA courses and became valuable teaching resources for achieving the learning objectives. The content was introduced and practiced using the activities presented in the teaching materials. When designing the content for the three courses, emphasis was placed on beef and dairy cattle, swine, and poultry (laying hens, broilers, and turkeys), since these represent the main animal species that contribute to the economy of Texas and North Carolina, where the collaborating institutions are located. A total of fifteen units were designed and distributed as follows: SSPA 1 comprised six units; SSPA 2 encompassed five units; and SSPA 3 included four units. As mentioned before, topics were selected based on the survey results, which evidenced their actual relevance in the agriculture industry. The last SSPA 3 unit was devoted to individual student presentations that focused on animal health, welfare, and food safety. Through these presentations, students were able to use the language orally as they addressed specific topics related to the three key areas mentioned above. Table 1 below displays the distribution of topics per unit and course.

#### 3.4.5. Contextualization

Contextualization was a key component of the overall design of the materials; this is also a key element in CLT and the constructivist approach to teaching. Contextualization is essential for achieving meaningful learning outcomes. For this reason, specialized vocabulary and the grammar items taught were derived from the contexts in which the conversations took place.

The topics assigned to the units included a brainstorming activity intended to introduce the topic, enhance student motivation, and explore students’ background knowledge for each unit. Then, specific situations closely connected to each topic were selected and conversations that were likely to take place in such situations were created. These conversations were tuned to match the students’ proficiency level. Each conversation allowed for the introduction of specific field-related vocabulary (key language items) and grammar, which were then practiced in further learning activities throughout the same unit. Simulated dialogs were pre-recorded for the conversations in SSPA 1 so that the students could be exposed to dialectal variations in the Spanish language. This was deemed important since students are expected to interact with Spanish-speaking caretakers from different Latin American countries in their jobs as professionals.

**Figure 1:**
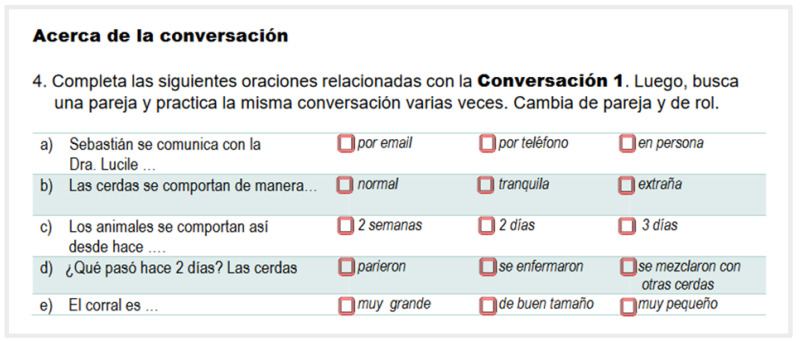
Example of a checking comprehension activity. “*Acerca de la conversación*” (About the conversation). Complete the following sentences related to **Conversation (1)** Then find a partner and practice the same conversation several times. (Exchange partner and role).

**Figure 2:**
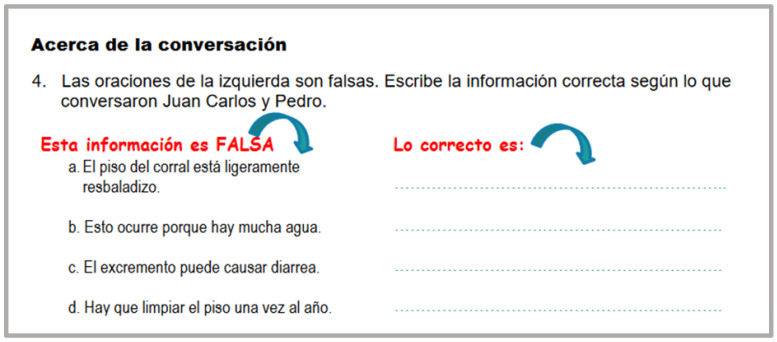
Example of a checking comprehension activity. “*Acerca de la conversación*” (About the conversation (4). The sentences on the right side are false. Write the correct information based on the conversation between Juan Carlos and Pedro).

**Figure 3:**
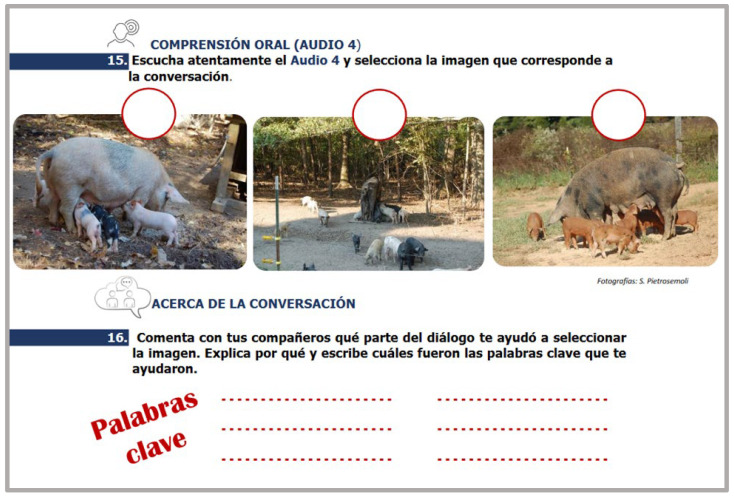
Example of a checking comprehension activity. “*Acerca de la conversación*” (About the conversation (15). Listen to Audio 4 carefully and choose the image that corresponds to the conversation (16). Discuss with your peer what section of the dialogue helped you choose the image. Explain why and write down the keywords that helped you find the answers).

Each conversation was followed by a checking comprehension activity called “*Acerca de la Conversación*” (About the Conversation). An effort was made to present these activities with a different and appealing layout even though their purpose remained the same. In this way, students’ motivational levels were kept high. Figure 1, Figure 2 and Figure 3 show examples of these activities.

**Figure 4:**
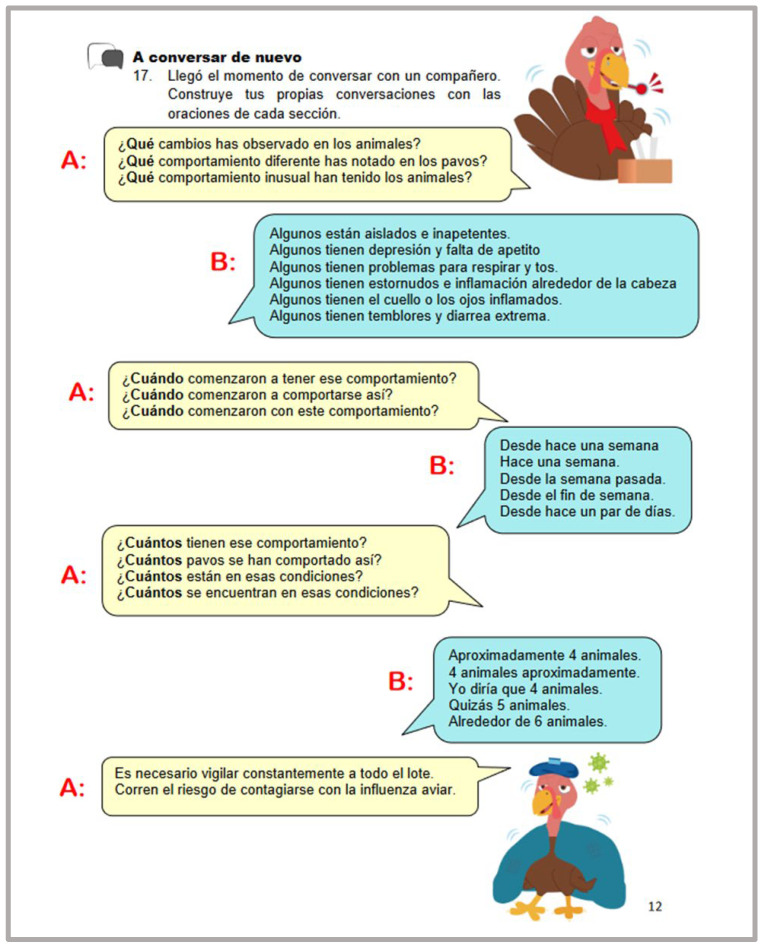
Example of an interaction activity (17): (It is time to speak with a partner. Create your own conversations using sentences from each section.).

**Figure 5:**
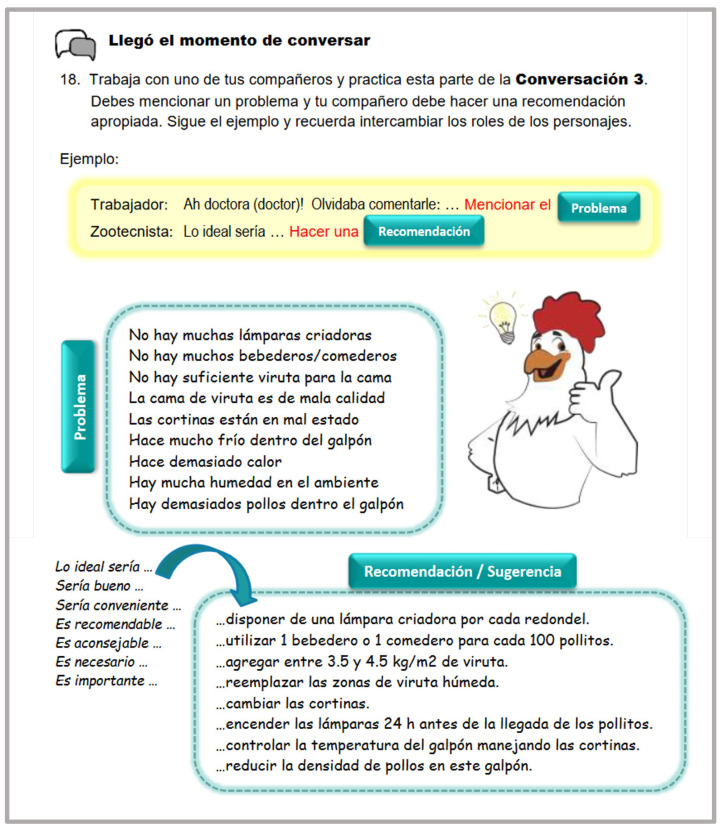
Example of an interaction activity. (It is time to talk: (18) Work with one of your partners and practice this section in Conversation 3. You must state a problem, and your partner should provide an appropriate recommendation. Follow the example and remember to switch roles).

Students were provided with plenty of opportunities to interact in the target language. At the initial learning stages, guided interactions with strong scaffolding took place. The language instructor was always there to provide the necessary assistance and support. These interactions were mainly based on the content presented in the introductory conversations. Interactions continued to take place as students progressed to higher proficiency levels; however, these interactions had less scaffolding and the instructor’s assistance was less required at these stages, promoting the learner’s autonomy (Figure 4 and Figure 5). 

Other instructional resources that intended to expose the students to authentic input in Spanish were videos. Specialists in the species selected for this study were interviewed and recorded. The interviewees were asked to provide specialized information on their field. A set of questions was prepared in advance and then clearly and spontaneously answered by the specialists. Given the nature of the oral mode of the language, characterized by pauses, hesitations, shifts in ideas, and incomplete sentences, among others, the videos were edited to show a more coherent discourse without affecting their spontaneity and authenticity. A worksheet was designed comprising activities requesting general and specific information on certain aspects dealt with in the video. These audio-visual resources, along with the worksheets, were used by the students at their own pace as a complement to the teaching sessions. 

#### 3.4.6. Vocabulary

**Figure 6:**
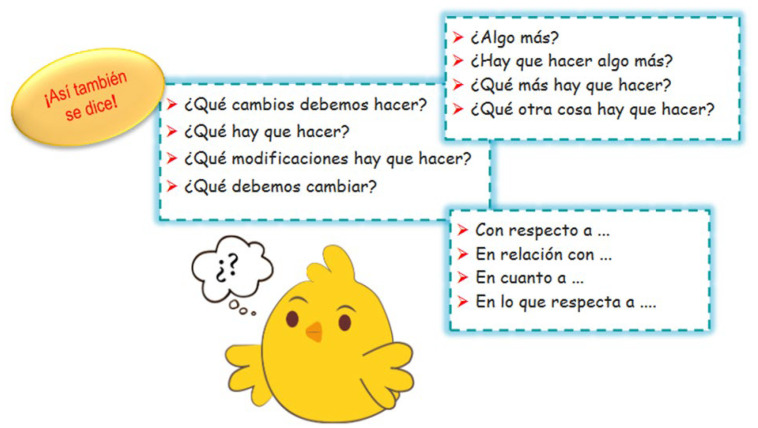
Example of “Asi Tambien se dice” (you can also say it this way).

Vocabulary acquisition was addressed through a section labeled “*Así también se dice*” (You can also say it this way). This section presented either synonyms or equivalent phrases to the key vocabulary in the conversations. This aimed to provide students with opportunities to expand their vocabulary while they roleplayed the initial conversations and replaced the key vocabulary. As they engaged in these further roleplays, they became more skilled in the use of the new vocabulary and specialized terminology (Figure 6).

**Figure 7:**
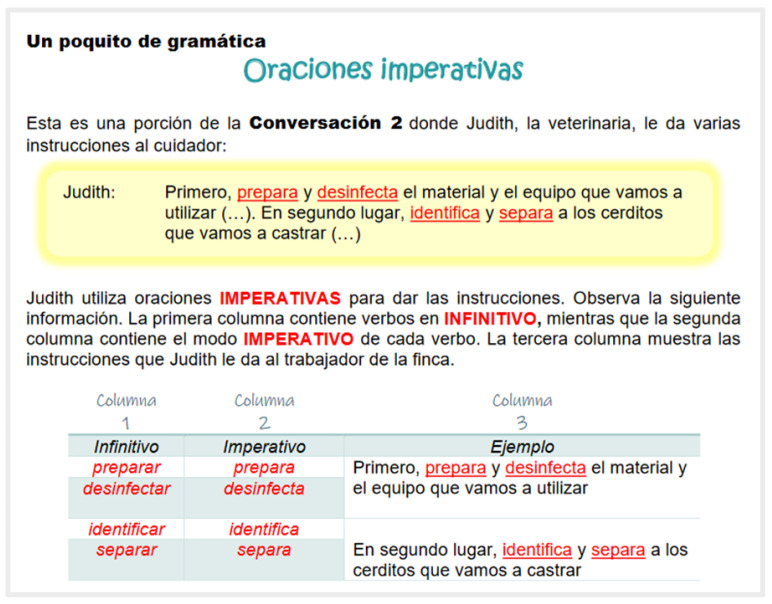
Example of “*Un poquito de gramática*” (A little bit of grammar. Imperative sentences).

The units also included a grammar section named “*Un poquito de gramática*” (A little bit of grammar). This section presented excerpts of the initial conversations where a specific aspect of grammar was introduced. In this way, the grammar was approached in a contextualized manner, prompting the students to make their own inferences and to become aware of the function or purpose of that grammatical point. Formal explanations were then provided, and written exercises were provided for students to further practice each aspect of the grammar (Figure 7).

**Figure 8:**
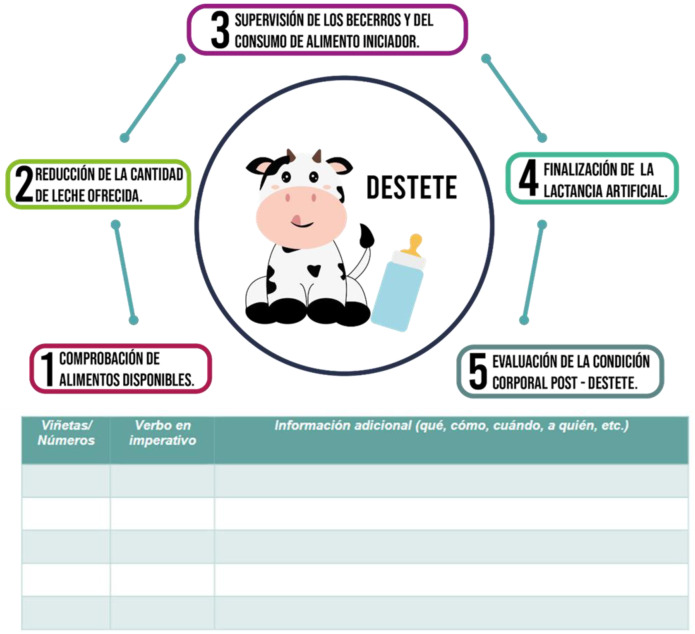
Example of illustration:(30). Take a look at the illustration and complete the table below.

**Figure 9:**
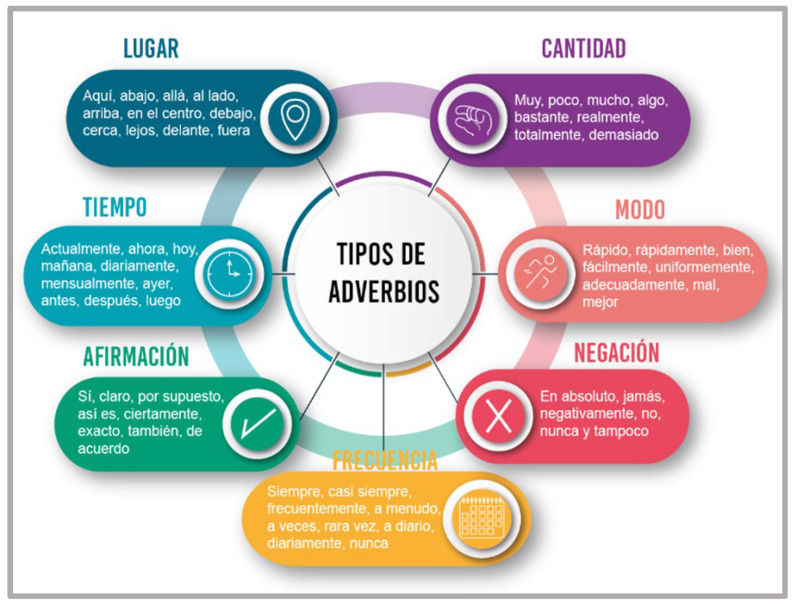
Example of illustration (Types of adverbs).

**Figure 10:**
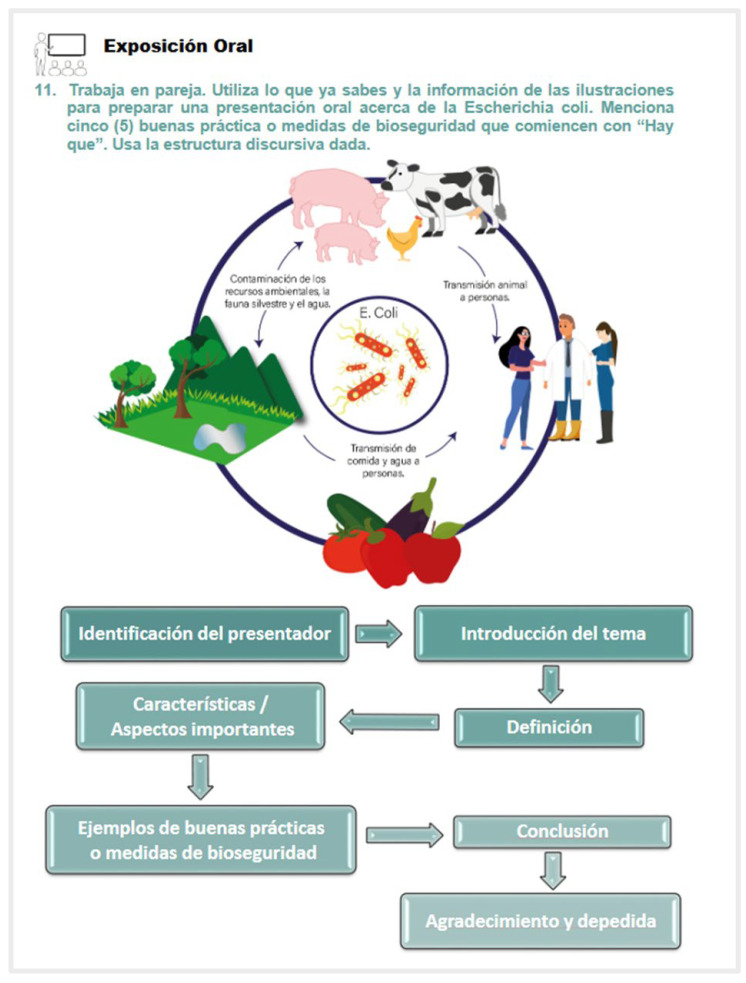
Example of illustration. (Oral presentation (11) Work in pairs. Use your prior knowledge and the information on the illustration to create an oral presentation on *Escherichia coli*. State five best practices or biosecurity measures beginning with “Hay que”. Use the discurse structure given below).

The materials also included pictures, illustrations, and other visual aids such as graphic organizers, concept maps, and diagrams. The pictures presented realistic information associated with the topics dealt with in conversations, which, in turn, provided contextualization. In addition to making the materials more appealing to the students, the illustrations and cartoons contributed to the creation of a pleasant teaching environment as they promoted a less formal academic atmosphere and helped to lower stress and anxiety levels. Using the graphic organizers, concept maps, and diagrams, students were able to transcode information from the written language presented as the input and vice versa. These resources were also used as a starting point for discussions (Figure 8, Figure 9 and Figure 10).

#### 3.4.7. Metacognitive Strategies

**Figure 11:**
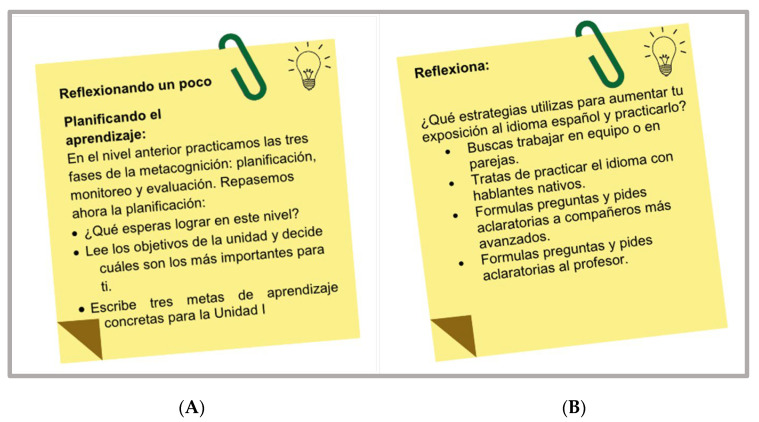
Example of metacognitive reflection (**A**,**B**).

**Figure 12:**
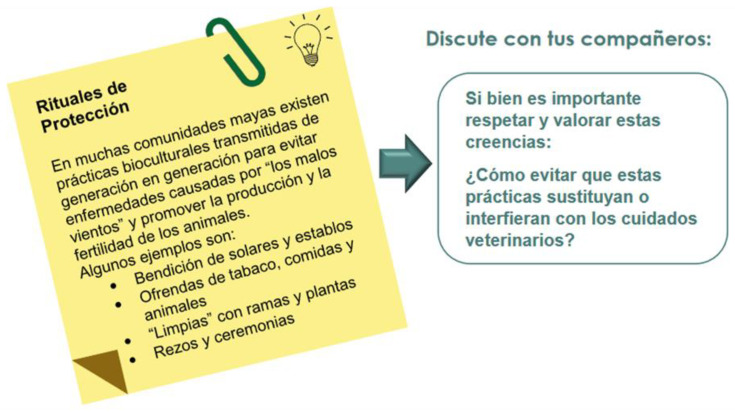
Example of cultural competencies.

An innovative feature of the series of SSPA materials reported in this paper was the integration of metacognitive strategies. Each of the units displayed a set of sticky notes or posts containing statements or questions intended to make the students reflect upon their own learning process. Such metacognitive strategies served to gradually increase students’ awareness of how they learn and provided valuable tools for planning, assessing, and enhancing their learning experiences (Figure 11). Metacognitive strategies were also used to approach the cultural aspect of the foreign language by presenting and prompting the students to reflect upon specific situations that are part of the culture of countries where the Spanish language is spoken (Figure 12).

#### 3.4.8. Challenges and Adjustments

During the implementation phase, several challenges were encountered, including technical issues with the online platform and the need to balance the course content with the students’ existing academic commitments. These challenges were addressed through adjustments to the course schedule, additional technical support, and iterative refinements to the course materials based on student feedback. Despite these challenges, the overall outcome of the courses was positive, demonstrating the value of tailored Spanish language instruction for veterinary and animal science students and professionals.

### 3.5. Implementing the Courses

#### 3.5.1. Spanish Placement Exam

**Figure 13:**
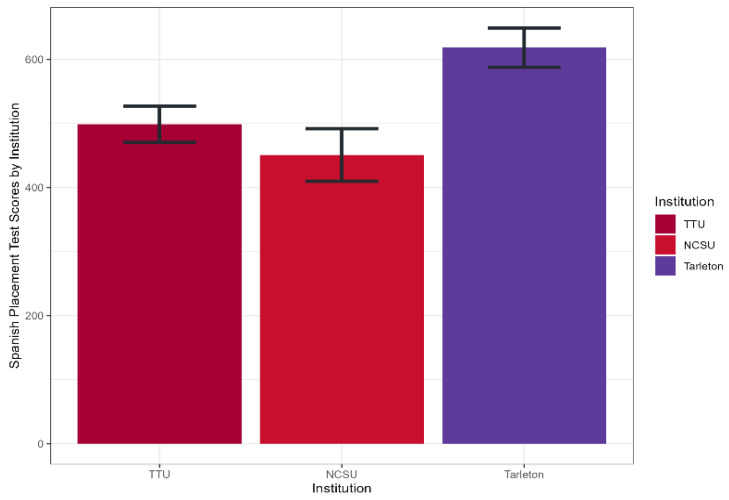
Spanish placement exam scores by institution.

The normality of the residuals was *p* > 0.05, suggesting that the residuals followed a normal distribution. For the homogeneity of variances, the *p*-value was 0.01, indicating that the variances were homogeneous and supporting the validity of the ANOVA results. Students who participated in the program obtained an average score of 522.78 out of 850 points in the Spanish Placement Exam. After analyzing variance (ANOVA) to examine differences between institutions, significant differences in scores (F (2.86) = 4.543, *p* = 0.01) were found. These results suggest that the students’ institution significantly affected their placement test scores (Figure 13). Tukey’s post hoc multiple comparisons and Bonferroni Test analysis further revealed significant differences between TSU and NCSU (*p* = 0.01) and between TSU and TTU (*p* = 0.04), while no significant differences were found between TTU and NCSU (*p* = 0.56). These comparisons suggest that TSU students performed better than NCSU students in the Spanish Placement Exam, with a marginally significant difference in performance compared to TTU.

#### 3.5.2. Student Commitment

A total of 146 students expressed an interest in enrolling in the program. Out of these, 89 students completed the TTU Spanish Placement Exam (SPLEX) and 69 (77.52%) passed, with an average score of 586. Twenty students (22.47%) did not pass the test, obtaining an average score of 255.2. 

Of the 69 students who initially passed the SPLEX and were admitted to the program, 32 (46.37%) withdrew while 37 (53.62%) successfully completed all three courses and met the program requirements. Dropouts only occurred in SSPA 1 (46.37%). 

#### 3.5.3. Student Performance in the SSPA Courses

**Figure 14:**
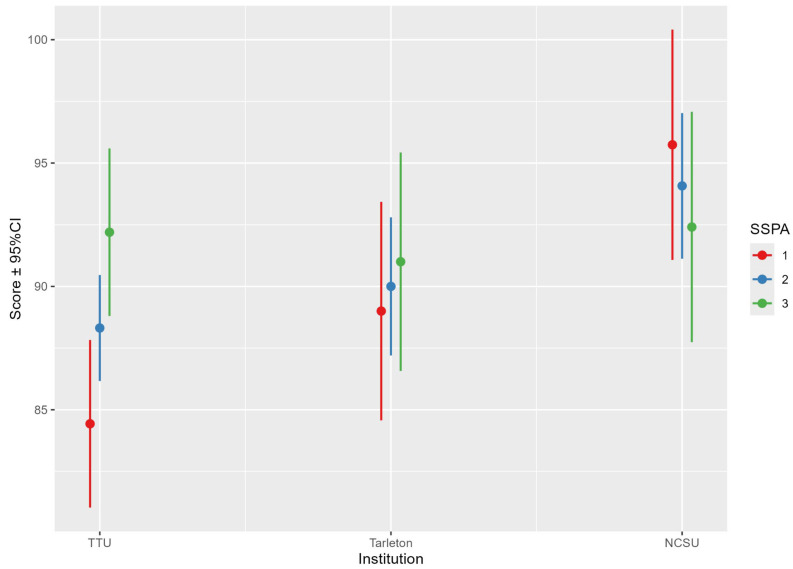
Interaction between institution and final SSPA scores across three courses.

A parametric repeated measures ANOVA was performed to assess the final scores across the three SSPA courses. The results indicated that the interaction between institution and SSPA scores was statistically significant, *p* = 0.02, suggesting significant variation in SSPA scores across institutions (Figure 14). Mauchly’s test indicated a violation of sphericity (*p* < 0.001), so Greenhouse–Geisser corrections were applied. This effect remained significant after applying the Greenhouse–Geisser corrections (ε = 0.747, *p* = 0.03).

A marginal means analysis was conducted to evaluate participant performance across the three institutions (TTU, NCSU, TSU) under three conditions (SSPA = X1, X2, and X3). It is important to note that the marginal means reported are adjusted values derived from the statistical model. These adjusted means account for covariates and other factors included in the analysis, and as such, they represent predicted averages rather than raw scores. Consequently, the marginal means may slightly exceed the maximum possible raw score due to the model’s adjustments and predictions (Table 2).

These findings indicate that the estimated means of the final test scores of NCSU students were consistently higher than those obtained by TSU and TTU students across all the courses, particularly in SSPA1.

#### 3.5.4. Correlation Analysis

**Figure 15:**
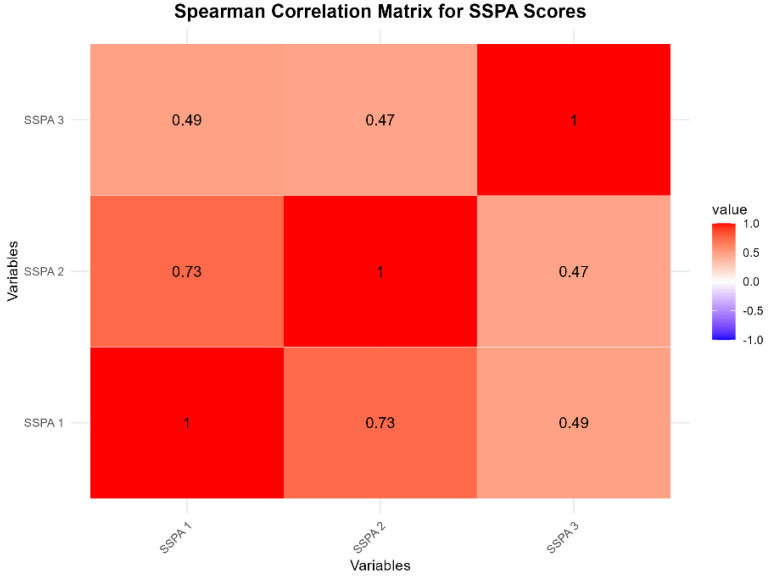
Correlation matrix between SSPA 1, 2, and 3 performance scores.

A heatmap analysis (Figure 15) shows a strong positive correlation (r = 0.73, *p* = 0.0001) between performance in SSPA 1 and SSPA 2, indicating that students who performed well in one course tended to perform well in the other. Similarly, a moderate positive correlation was observed between SSPA 1 and SSPA 3 (r = 0.49, *p* = 0.0023) and also between SSPA 2 and SSPA 3 (r = 0.47, *p* = 0.0032), demonstrating that all of the scores are also related in the same direction. 

## 4. Discussion

The demand for Spanish for Specific Purposes in Agriculture (SSPA) university courses in the U.S. has increased significantly because of the need for professionals to effectively communicate with Spanish-speaking workers in specific fields. This need is particularly pronounced in agriculture, where the number of Hispanic workers is continuously growing [39]. Three SSPA courses were developed to address these communicative needs. The course design was informed by a survey exploring the language needs of non-Spanish-speaking professionals who face communication difficulties in their work environments. The survey results highlighted the importance of mastering Spanish oral skills for understanding descriptions of animal conditions, behavioral changes, disease symptoms, animal management protocols, treatment administration, humane handling, euthanasia, and record-keeping [9]. Communication barriers between English-speaking professionals and the Hispanic workforce can be mitigated by focusing on oral receptive (listening) and oral productive (speaking) skills, with less emphasis on written skills. The courses used a blended teaching approach that integrates communicative language teaching, constructivist theory, and metacognitive strategies. This comprehensive approach aims to maximize students’ cognitive potential and provide a rich learning experience. Our study indicates that this approach effectively bridges communication gaps between prospective animal professionals and Spanish-speaking animal caretakers. Teaching materials, resources, and strategies were designed to create an enriched learning environment, emphasizing Spanish as both the goal and the means of communication. This allows learners to develop their language skills through cooperative interactions in realistic farm contexts. These findings suggest that tailored SSPA courses can address the specific issues and needs identified by experienced professionals. Unlike conventional language instruction, SSPA courses focus on animal health, animal welfare, and food safety. This field-oriented approach ensured that the students developed the necessary oral and written communication skills to effectively use Spanish in their work settings. 

The analysis of student commitment was different from the one reported by Sánchez-López [22], in which lower student engagement was reported. Their study showed that 86 students were enrolled over five years and that only 27 students (31.39%) completed the program while our program showed a completion rate of 53.62% in one and a half years. Although our program evidenced a higher completion rate, the continued refinement of course delivery and support structure should always be considered. 

Teaching and learning are, in essence, multifactor processes for a set of elements that must come together and interrelate coherently so that the learning goals can be achieved. Key components such as teaching strategies and procedures, student-centeredness, meaningful learning, active communication, teaching mediation, teaching materials and resources, contextualization, and metacognitive processes were carefully selected and harmoniously assembled to bridge the communication gap previously identified. Students were actively engaged in constructing their knowledge and acquiring the target language, with the instructor providing essential support through a robust scaffolding system. The course materials and resources were tailored to the communication needs reported in the survey, considering the learners’ prior knowledge and experiences. This contextualized approach helped learners understand the purpose of using the target language and facilitated the development of communicative competence in Spanish. Participants not only improved their Spanish skills, but also gained relevant professional competencies, such as understanding protocols and One Health concepts.

Both the commitment and completion rates among veterinary and animal sciences students in specialized courses like SSPA can be affected by several factors. Although these factors were not explored, the commitment rate in our study was higher than that reported by Sánchez-López [22]. Another factor that can be associated with the completion rate is that our SSPA courses did not offer any credit, which probably led to students perceiving the course as less relevant. On the other hand, Colorado State University [33] did include credits for their Spanish courses, which may have contributed to student engagement. Another important factor to consider here is the heavy academic load that veterinary programs have. Such programs are typically demanding; therefore, students might struggle to balance the requirements of their core veterinary courses with the additional work demanded by the language courses, making it challenging to incorporate synchronic classes into their busy schedules. In this sense, our experience with the SSPA courses resembles that of Zeller, Frye, and Frey [33]. Some of our SSPA students struggled with course schedules and even though several options were provided to overcome this issue, some of the students ended up dropping the courses, indicating that none of the implemented alternatives were enough to support their commitment. 

To be enrolled in the program, candidates were required to have a minimum SPLEX score of 300 showing that they had prior knowledge of basic Spanish, either because they had received previous formal instruction or because they had acquired language skills in multicultural family settings. Despite their basic competence in Spanish, they lacked knowledge of specific terminology pertaining to the agriculture industry. Our experience in the implementation of the SSPA program showed that participants were not only able to master aspects related to the target language but also gained the ability to communicate specific content using the specialized vocabulary in the framework of selected topics dealing with animal husbandry, production, health, welfare, biosecurity, and food safety.

The observed significant differences in performance outcomes (measured as final SSPA test scores) across institutions can, in part, be attributed to differences in baseline Spanish language proficiency. The results of the Spanish Placement Exam revealed that TSU students performed significantly better than their peers from NCSU and TTU before starting the program. This indicates that TSU students began the SSPA courses with a stronger foundation in Spanish, which likely facilitated their performance in their courses. Although all three institutions utilized the same instructor and course materials, other factors may have contributed to the observed differences in baseline proficiency and course performance. While demographic data from the students were not collected, the Spanish Placement Exam functions as a measure of students’ prior exposure to the language. TSU students may have had more access to Spanish instruction during their education or may have been exposed to Spanish-speaking environments, providing them with an advantage in the SPLEX. TSU might have a higher proportion of students from regions with strong bilingual or Spanish-speaking communities, offering students additional learning opportunities outside the classroom.

Significant differences in performance outcomes (measured as final SSPA test scores) among the institutions and across the different courses were identified. When institutions were compared, significant differences (*p* < 0.05) were found between the means of the final test scores for SSPA 1, SSPA 2, and SSPA 3. The statistical analysis of student performance and the correlation analysis provide quantitative support for the efficacy of the SSPA courses. The positive feedback from students and the observed progress in language acquisition reaffirms the value of tailored language instruction in veterinary and animal science contexts.

Although many factors impact language learning [35,36,37,38], teaching strategies have been identified as the most investigated factor [36]. Given that all of our SSPA students were exposed to the same teaching strategies, as well as other external factors that influence language acquisition, such as the instructor, teaching materials, language input, and classroom procedures, it is likely that the differences in student performance (average final scores per course) among the participating institutions are the result of a combination of other influencing factors of an individual and social nature. However, the influence of such factors was not the focus of attention in our work. 

Communication challenges with Spanish-speaking animal caretakers depend in part on how current and future veterinary and animal science students are trained; therefore, developing SSPA courses tailored to the participants’ particular needs is critical for their professional performance. The successful implementation of the SSPA courses reported in this paper underscores the importance of tailoring language education to specific professional needs. These courses can be replicated or adapted to other university audiences aiming to develop Spanish language skills for effective interactions with Hispanic workers in agricultural settings. As the demand for SSP courses is expected to grow, studies like ours provide valuable insights into the design of effective teaching procedures that can enhance the competitiveness of the U.S. agriculture industry. 

## 5. Conclusions

The increasing number of Hispanic animal caretakers who are monolingual presents a unique challenge for English-speaking veterinarians and animal science professionals. These experts must not only communicate in Spanish, but a specialized form of Spanish based on agricultural terminology. Addressing communication barriers between animal professionals and the Hispanic workforce is crucial for promoting animal health and welfare, food safety, and the sustainability of U.S. agriculture. Therefore, developing formal courses tailored to the needs of the livestock industry is essential to ensure that students learn content that is both relevant and applicable on the farm. Our work demonstrates that it is possible to create industry-specific content to improve communication between veterinarians, animal scientists, and the Hispanic workforce. 

All the students enrolled in the SSPA courses fulfilled the entry requirement of passing the SPLEX. However, each SSPA course contained students with different proficiency levels. Students with a Hispanic background exhibited more daily live communication competence than those who were trained in formal educational settings. Nevertheless, the average scores across the three courses were above 88, indicating that students were able to understand and process the content effectively. Moreover, all of the students benefited from the courses in the sense that they were able to acquire specialized terminology of the field and engage in technical conversations in a more accurate, fluent, and confident manner. This supports the idea that the SSPA courses are relevant when training prospective animal professionals and preparing them to effectively deal with the challenges imposed by communication barriers in agriculture work scenarios. Improvements such as offering supplemental resources and tutoring are deemed important for less proficient students who may experience difficulties with the course materials. Although this work focused specifically on bridging the communication gap in agricultural settings, it can be used as a model to bridge communication gaps in veterinary clinic settings where veterinarians must communicate with Spanish-speaking clients.

## Figures and Tables

**Table 1:** Distribution of topics per unit and course.

Course	Unit	Topics
**SSPA 1**	1	*Conociendo algunos animales para el consumo humano* (Learning about some food animals)
	2	*Medio ambiente y producción animal* (Environment and animal production)
	3	*Prácticas de manejo para la cría de animales* (Management practices for animal husbandry)
	4	*Sanidad y bienestar animal* (Animal health and welfare)
	5	*Bioseguridad* (Biosecurity)
	6	*Seguridad alimentaria* (Food safety)
**SSPA 2**	1	*Buenas prácticas de manejo* (Management practices)
	2	*Alimentando a los animales de la granja* (Feeding farm animals)
	3	*Manejando la salud animal* (Managing animal health)
	4	*Actividades en la granja* (Activities on the farm)
	5	*Salud e higiene de los operarios* (Health and hygiene of the workers)
**SSPA 3**	1	*Manejo del parto distócico en bovinos* (Management of dystocic birth in cattle)
	2	*Manejo en producción de aves ponedoras* (Management in laying bird production)
	3	*Manejo de porcinos pre-faena* (Pre-slaughter pig management)
	4	Students’ presentations focused on animal health, welfare, and food safety.

**Table 2:** Marginal mean analysis.

SSPA	1			2			3		
Institution	TTU	NCSU	TSU	TTU	NCSU	TSU	TTU	NCSU	TSU
Mean	90.8	100.5	96.2	90.1	95.4	91.9	94.9	95.1	94.1
Std. Error	5.75	6.75	6.75	2.71	3.18	3.18	3.43	4.03	4.03
CI 95%	79.0–102.5	86.7–114.2	82.5–110.0	84.6–95.6	88.9–101.8	85.4–98.3	87.9–101.9	86.9–103.3	85.9–102.3

Degrees of freedom 33.

## Data Availability

All data included in the manuscript and Supplementary Materials are available at Garcia’s Laboratory.

## Supplementary Materials

The following supporting information can be downloaded at: https://www.mdpi.com/article/10.3390/ani14243639/s1, File S1: Flyer used to recruit the students and the reviewers.
