# Supplementary figures and images for: Developing Courses of Spanish for Specific Purposes in Agriculture to Bridge the Communication Gap Between the Hispanic Workforce and English-Speaking Veterinary and Animal Sciences Students
Source: Animals (Basel). 2024 Dec 17;14(24):3639. doi: 10.3390/ani14243639 (PMC11672768; doi:10.3390/ani14243639)

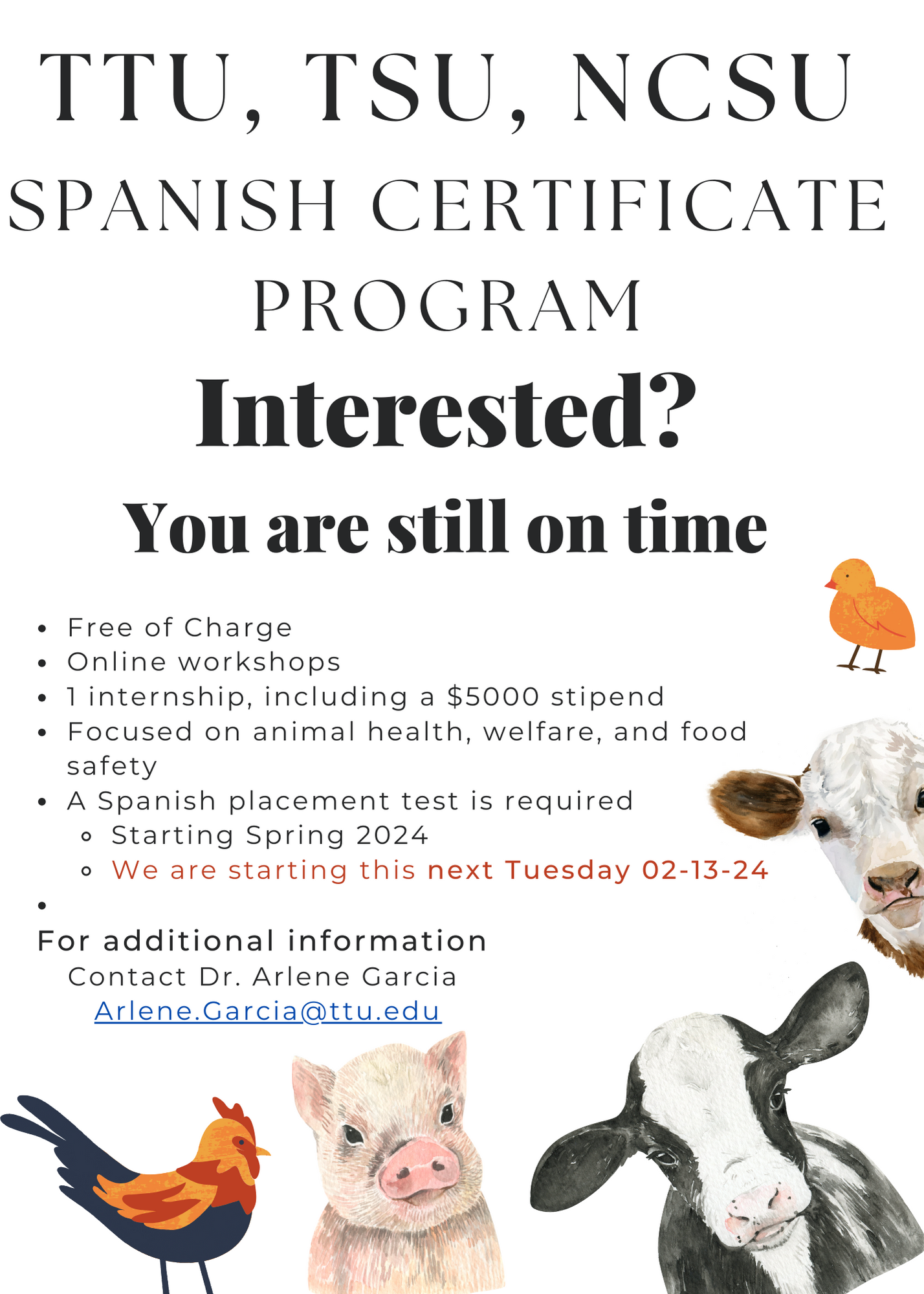

Supplement: Supplementary file 1 [file animals-14-03639-s001.zip › animals-3327356-supplementary.png]
